# Exploring the role of identity in pro-environmental behavior: cultural and educational influences on younger generations

**DOI:** 10.3389/fpsyg.2024.1459165

**Published:** 2024-10-09

**Authors:** Steffen Wild, Lydia Schulze Heuling

**Affiliations:** ^1^Department of Rehabilitation Sciences, Technical University Dortmund, Dortmund, Germany; ^2^Western Norway University of Applied Sciences, Bergen, Norway

**Keywords:** cooperative students, Germany, personal identity, pro-environmental behavior, self-categorization theory, social identity

## Abstract

It is of paramount importance to gain an understanding of pro-environmental behavior if we are to successfully tackle the climate crisis. The existing body of research provides evidence that identity influences pro-environmental behavior. However, such research is often over-generalised and researchers are challenged to conduct robust analyses with regard to specific local, cultural and educational factors. The present study aims to investigate whether personal or social identity has a distinct effect on three different dimensions of pro-environmental behavior, using the principles of self-categorisation theory. Additionally, the study seeks to determine whether one of these two factors, the individual or the social factor, is predominant over the other. The study group consisted of cooperative students in Germany, typically a group with high professional ambitions. The data was collected in a cross-sectional survey with a total of 568 cooperative students from academic disciplines in engineering and economics. The reliability of the scales is satisfactory (ω = 0.76–0.88), and the hypotheses are tested by estimating structural equation models. Our research demonstrates that while social identity exerts a stronger influence on activist behavior than personal identity, personal identity has a more pronounced effect on consumer behavior than social identity. Nevertheless, no general statement can be made regarding the relative strength of the effects of personal and social identity on pro-environmental behavior dimensions.

## Introduction

The current climate change emergency is associated with a range of threats to individuals, social groups and the natural environment. These include poorer mental health, violence and increased temperatures (Cianconi et al., 2020; Clayton et al., 2014; Ferguson and Schmitt, 2021; Hsiang et al., 2013; Levy et al., 2017). A number of academic disciplines, including psychology, sociology, political science and education, are investing in research in this area (Dono et al., 2010). Pro-environmental behavior (PEB) has been identified as a key topic in the academic literature (Lange and Dewitte, 2019; Martin et al., 2020; Steg and Vlek, 2009).

Researchers have tried to understand the factors associated with PEB. These include. identification with nature (Mackay and Schmitt, 2019), guilt (Hurst and Sintov, 2022), morality (Schmitt et al., 2019) and threat (Schmitt et al., 2019). There are also analyses of connections between connectedness to nature and its impact on sustainable behavior and happiness in children (Barrera-Hernández et al., 2020). Further research has identified a correlation between identity and PEB (Fritsche et al., 2018; Mackay et al., 2021). However, in the aforementioned field of research, there is a debate about the distinct impact of individual and social identity on PEB (Fielding and Hornsey, 2016; Hoppe et al., 2023; Udall et al., 2021). PEB is predominantly conceptualized in a one-dimensional, highly generalized manner (Mateer et al., 2022), and an analysis of the population fails to consider cultural and educational background sufficiently (Kirbiš, 2023; Mónus, 2022).

The objective of our research is to investigate the challenges described with the aim of expanding knowledge and providing new insights. In particular, we develop a distinct view of personal and social identity and their association with PEB. We shed light on three dimensions of PEB by distinguishing between willingness to pay, consumer behavior and activist behavior. In this research, we invest in cooperative students from Germany. The number of participants in this educational program in higher education has increased from approximately 64,093 in 2012 to 120,517 in 2022 (Hofmann, 2023). Students alternate every 3 months between work experience in a company with a contract and studying at university (Wild and Neef, 2019; Wild and Neef, 2023), while enrolling in a demanding and popular academic discipline of economics and engineering (Federal Statistical Office of Germany, 2022, p. 31). The program is regarded as a “selection of the best” through practice (Kupfer, 2013; Weich et al., 2017). Furthermore, students engaged in cooperative education are more likely to be from a lower socio-economic background than those enrolled in conventional higher education programs (Kramer et al., 2011). Germany is in a unique position, as it has one of the largest economies in the world (Gündüz et al., 2022). Furthermore, all nuclear power plants have been shut down in recent years, and the Green party currently holds a leading role in the German government.

### Self-categorization theory and pro-environmental behavior

The 13th UN Sustainable Development Goal, ‘Climate Action’ (SDG; United Nations, 2022), posits that PEB efforts are necessary to combat human-induced climate change (Bauernschmidt et al., 2023; Lange, 2023; Rau et al., 2022). However, there is no consensus regarding the definition of PEB. Researchers have proposed that PEB is understood as “behavior that consciously protects the environment and improves its sustainability” (Tian and Liu, 2022, p. 2). The identity framework is employed to elucidate the concept of PEB (Fielding and Hornsey, 2016; Fritsche et al., 2018).

The approach of Social Identity Theory (SIT; Tajfel, 1978) and its further development of Self-Categorization Theory (SCT; Turner, 1999) is used to show associations as well as explanations with PEB (Fielding and Hornsey, 2016; Mackay et al., 2021; Rabinovich et al., 2012; Trepte and Loy, 2017). Schulte et al. (2020) explain the starting point of these frameworks by asking why members of low-structured groups with the same values and emotions act together to change their disadvantage in the social system (Tajfel, 1978). In SIT, a collective identity to a group and resulting actions are created through belonging to a group, seen as “in-group” and us, and through a positive conotated against other groups and their members, seen as “out-group” and them (Islam, 2014; Schulte et al., 2020). Group membership is a central feature when explored in PEB, it further influences the perception and evaluation of environmentally relevant issues, but also the extent to which we act sustainably (Reese et al., 2018). In other words, the likelihood of group members making pro-environmental decisions increases when there are high norms for PEB in their group. It is also worth mentioning that (1) people belong to different groups, (2) people tend to behave as they think their group should behave, and (3) there is a social comparison with another group in terms of higher or lower social status, which influences people’s decisions in group membership (Sakala et al., 2021). This approach has advantages as it explains (1) a focus on group membership encourages (or discourages) investment in addressing particular environmental problems and (2) an understanding of ingroup-outgroup relationships is an influential factor in progressing environmental policy (Fielding and Hornsey, 2016).

The SIT assumption suggests a continuum of interpersonal and intergroup behavior (Trepte and Loy, 2017). In contrast, SCT supports the person as an individual. In SCT, social and personal identity processes operate simultaneously.

### The influence of personal identity on pro-environmental behavior

Personal identity refers to self-descriptions in terms of personal and idiosyncratic attributes (Kish Bar-On and Lamm, 2023; Turner et al., 1987). People see themselves as ‘I’ and ‘me’ (Haslam et al., 2022). Consequently, this sets a person apart from others. Thus, people experience themselves as unique individuals (Haslam et al., 2022; Turner, 1982). In SCT, people create their own judgmental decision-making process. In other words, the identity of the self is done actively and interpretatively, reflecting an interaction through own motives, own expectations, own knowledge and reality (Trepte and Loy, 2017; Turner, 1999).

According to Zacarés and Iborra (2015), personal identity is considered as an aspect of self-definition at the individual level through goals, values, beliefs and a whole set of related self-representations and self-evaluations. This background allows us to identify empirical associations between personal identity and PEB. Researchers use related frameworks, such as environmental self-identity (van der Werff et al., 2014a). In line with Udall et al. (2021), we consider environmental identity as individual identity and social identity as group identity.

Empirical evidence suggests that environmental self-identity is related to PEB in a variety of ways. There are specific and general analyses of these constructs that support this. Analyses by Shadiqi et al. (2022), using a cross-sectional design of 202 university students, and van der Werff et al. (2021), recruiting 2,479 individuals through the newsletter of electric vehicle charging company New Motion in the Netherlands in 2017, reported associations between general environmental self-identity and PEB. Further research demonstrates the association between general environmental self-identity and specific PEB of washing with a full load, turning off lights, turning off appliances, setting temperature during the day, setting temperature at night (van der Werff et al., 2021) using a sample of 4,796 persons in Italy, Switzerland and the Netherlands, as well as reducing meat consumption, product choices and judging environmental dilemmas by 150 students at a Dutch university who participated in exchange for course credits (Van der Werff et al., 2014b). Gaterslebena et al. (2014) show an association between environmental and thrifty identities on general PEB using a random sample of 135 households in a northern city and a southern city in the UK. In a deeper analysis, Gaterslebena et al. (2014) list robust associations from attitudes and personal norms on avoiding car use for shopping, avoiding car use for work, not flying to holiday destinations, buying fair trade coffee and tea, and recycling using a sample of 2,293 participants in different countries across Europe. Ruepert et al. (2016), using a sample of 618 employees in four countries in Europe, show that personal norms for environmentally friendly behavior at work are associated with lower energy consumption, higher transport-related energy consumption, higher transport-related energy savings, waste prevention at work and recycling.

### The influence of social identity on pro-environmental behavior

Social identity is concerned with self-definition in terms of group membership (Kish Bar-On and Lamm, 2023; Turner et al., 1987). Social identity is developed through a deductive process from the higher order group level. Central determinants are identification and norms through a separation of how ‘we’ can distinguish ‘us’ from ‘them,’ where ‘we’ is seen as a positive differentiation (Jans, 2021; Turner et al., 1987). Comparing personal and social identity, Crocetti et al. (2018) summarize that personal identity illustrates structures and processes belonging to one’s own identity, whereas social identity sheds light on motivational and social cognitive processes of one’s own identity.

Looking more closely at social identity, researchers separate cognitive, evaluative and commitment components (Hehir et al., 2021). The cognitive component, or self-categorization, is concerned with belonging to a group based on similarities with group members or differences from people outside the group (Algesheimer et al., 2005; Ellemers et al., 1999). The evaluative component takes into account the value aspect of belonging and being associated with a group, such as a sense of belonging, identification, expectations, having a place there, and group self-esteem (Ellemers et al., 1999; McMillan and Chavis, 1986). The commitment component is based on emotions and feelings of being a group member (Bagozzi and Dholakia, 2006).

Empirical findings support the association of social identity with PEB in a variety of ways. Hehir et al. (2021) show an association of general social identity on general PEB using a sample of 217 students under the age of 18 in a youth-based program. Research by Chumg et al. (2019) supports this finding using a sample of 548 Chinese employees from Jiangsu province. Further research by Schulte et al. (2021) provides further evidence to explain the process between social identity and overall PEB participation intention, using indirect effects via group-based emotions and collective efficacy. Dono et al. (2010) provide a detailed analysis and identify associations of general social identity on three PEB of environmental citizenship, consumer behavior and willingness to pay based on a sample of 131 students from an Australian university.

### An overview of whether personal or social identity has a stronger influence on pro-environmental behavior

The field of personal and social identity has only recently begun to be explored through competing analytical frameworks. Despite the numerous existing studies on identity, the initial analyses were conducted separately for each construct. Consequently, the interrelationship between personal and social identity is regarded as a relatively under-researched area (Crocetti et al., 2018).

Theoretical assumptions provide a clear position regarding the interplay between personal and social identity. In the context of the Social Identity Theory (SIT), Turner et al. (1994) posit that personal and social identity are equally valid and authentic for the expression of the psychological process of self. Consequently, personal and social identity should contribute equally to the understanding of the process by which individuals come to understand themselves and their place in society.

However, empirical findings show the opposite. Using a sample of Dutch (*N* = 161) psychology students and Chinese (*N* = 168) business students, Wang et al. (2021) show that personal-level variables and PEB have a stronger effect than group-level variables in all subsamples. Further research through meta-analyses supports such findings. Vesely et al. (2021) find a stronger effect of environmental self-identity on PEB variables of intentions and behaviors (*r* = 0.62/0.56) than identification with groups perceived to support climate-friendly behavior on PEB variables (*r* = 0.48/0.51). Udall et al. (2021), in further meta-analyses, show a stronger association of individual identities with overall PEB compared to group identities, but the researchers emphasize that the associations are stronger when they are in the same category (e.g., both are group-oriented).

### Objectives

As shown above, there is much evidence on the associations of personal and social identity on PEB. However, researchers have only been analyzing the comparison of the effect size between personal and social identity on PEB for a few years, as these two constructs were previously considered separate (Crocetti et al., 2018). Initial analyses show that personal identity should have a stronger influence on PEB compared to social identity (Udall et al., 2021; Wang et al., 2021; Vesely et al., 2021). However, recent research suggests that cultural, social and educational backgrounds have an influence on these associations (Kirbiš, 2023; Mónus, 2022). Consequently, there is a need for research on subpopulations for these associations, such as the cooperative education highlighted above. Here, we expect different effect sizes than in previous findings. On this basis, we formulate the following hypotheses.

*Hypothesis 1*: Higher personal identity with nature is positively associated with PEB.

*Hypothesis 2*: Higher social identity with nature will be positively associated with PEB.

*Hypothesis 3*: Social identity with nature shows a different effect size compared to personal identity with nature on the association with PEB.

In our research we focus on the cooperative education population. Here we are interested in the academic field of economics and engineering. We consider Germany to be a suitable country. The reasons for this are explained in the first chapter of the introduction above.

## Methods

### Participants and design

The data utilized in this study was derived from a follow-up investigation of the panel study, “Study Process - Crossroads, Determinants of Success and Barriers to Study at the DHBW” (Deuer and Meyer, 2020). This was conducted via a paper-and-pencil questionnaire in the spring of 2022. All cooperative students are enrolled in a Bachelor’s degree program at the Baden-Wuerttemberg Cooperative State University Ravensburg (DHBW). The study employs convenience sampling and a cross-sectional design.

A total of 568 participants were surveyed, with an average age of 21.18 years (*SD* = 2.08). The gender distribution of the sample was 117 female (20.6%), 448 male (78.9%), and three diverse students (0.5%). In our sample, 46% of respondents have at least one parent with a university degree. The academic discipline is economics for 150 participants (26.4%, field of study, industrial management and information systems) and engineering for 418 participants (73.6%, field of study, The disciplines of Electrical Engineering, Mechanical Engineering and Embedded Systems). A significant difference was observed in the distribution of students across academic disciplines, with male students tending to be more prevalent in engineering (80.8%) and female students tending to be more prevalent in economics (54.7%). This difference was found to be statistically significant (χ^2^ = 61.26, *df* = 2, *p* < 0.001, *Cramers’ V* = 0.33).

The study was approved by the Baden-Württemberg Cooperative State University (8 July 2015). At the outset of the study, the institution lacked an ethics committee. Consequently, the local heads of the research group assumed responsibility for ensuring ethical standards. Prior to the participants’ responses being obtained, informed consent was obtained and the anonymity of responses ensured. The study was conducted in accordance with the Declaration of Helsinki and the subsequent associated declaration (World Medical Association, 2013).

Prior to commencing the study, an *a priori* power analysis was conducted to determine the appropriate sample size. According to Westland (2010) and Soper’s (2022) software, a sample size of 200 participants with *r* = 0.30, power (1-β) = 0.80, α = 0.05, 15 manifest and three latent variables is sufficient to detect a significant result. Accordingly, the objective was to gather data from 500 individuals, anticipating that some participants’ data might be incomplete.

### Measures

The quality of the used instruments is analyzed by estimating the reliability of McDonald’s omega (McDonald, 1999). A value of ω ≥ 0.70 is deemed adequate (Viladrich et al., 2017). Furthermore, we estimated item difficulties as well as item-total correlation, with a result of *r_it_* ≥ 0.30 (Field et al., 2012, p. 803) and *p_i_* = 0.20–0.80 (Döring and Bortz, 2016, p. 477), which we consider to be encouraging. The instrument employs a 5-point Likert scale, ranging from 1 (strongly disagree) to 5 (strongly agree), or alternatively, from 1 (never) to 5 (always). All scales comprise three items. No new items have been developed for integration into the psychometric scales. Only original items from established scales are employed, with wording adapted for the population under investigation. The use of self-report measures may present a challenge due to the potential for participants to respond in ways that are not entirely truthful or socially acceptable (Demetriou et al., 2015). However, there is a school of thought (Wild et al., 2023) that asserts the use of self-reports yields robust results, akin to those observed in medical research (Short et al., 2009) and criminal investigation (Jolliffe and Farrington, 2014). For the sake of transparency, Table 1 provides a comprehensive list of all items used in both English and German.

**Table 1:** Scales and items of used measurement instruments.

Scale	Subscale	*N*		Item in English	Item in German
Social identity (Cameron, 2004)		3	SI1	I have a lot in common with other climate protester.	Ich habe mit anderen Klimaschützer*innen viel gemeinsam.
			SI2	I feel strong ties to other climate protester.	Ich fühle mich eng verbunden mit Klimaschützer*innen.
			SI3	I really “fit in” with other climate protester.	Ich passe wirklich gut mit anderen Klimaschützer*innen zusammen.
Environmental self-identity/Personal identity (Van der Werff et al., 2013)		3	ESI1	Acting environmentally-friendly is an important part of who I am.	Mich umweltfreundlich zu verhalten ist ein wichtiger Teil von mir.
			ESI2	I consider myself as a person that acts environmentally-friendly.	Ich bin eine Person, die umweltfreundlich handelt.
			ESI3	I see myself as an environmentally-friendly person.	Ich sehe mich selbst als umweltfreundliche Person.
PEB	Willingness to Pay (Stern et al., 1999)	3	WP1	I would be willing to pay much higher taxes in order to protect the environment.	Ich würde viel höhere Steuern zahlen, um die Umwelt zu schützen.
			WP2	I would be willing to accept cuts in my standard of living to protect the environment.	Ich würde Einschnitte in meinem Lebensstandard akzeptieren, um die Umwelt zu schützen.
			WP3	I would be willing to pay much higher prices in order to protect the environment.	Ich wäre bereit, viel höhere Preise zu bezahlen, um die Umwelt zu schützen.
	Consumer behavior (Stern et al., 1999)	3	CP1	How often do you make a special effort to buy paper and plastic products that that are made from recycled materials?	Wie oft bemühen Sie sich besonders, Papier- und Plastikprodukte zu kaufen, die aus Recyclingmaterialien hergestellt wurden?
			CP2	How often do you avoid buying products from a company that you know may be harming the environment?	Wie oft vermeiden Sie es, Produkte einer Firma zu kaufen, von der Sie wissen, dass diese die Umwelt schädigt?
			CP3	How often do you make a special effort to buy household chemicals such as detergent and cleaning solutions that are environmentally friendly?	Wie häufig geben Sie sich besondere Mühe, Haushaltchemikalien wie Reinigungsmittel und Reinigungslösungen zu kaufen, die umweltfreundlich sind?
	Activist behavior (Schmitt et al., 2019)	3	AB1	Got involved with a club or group whose main aim is to preserve and protect the environment.	Aktiv sein oder zu werden in einer Gruppe oder in einem Verein, deren oder dessen Hauptziel der Erhalt und der Schutz der Umwelt ist.
			AB2	Handed out fliers or put up posters in public locations to raise awareness about environmental issues.	An öffentlichen Orten Flyer verteilen oder Poster aufhängen, um das Bewusstsein für Umweltthemen zu steigern.
			AB3	Wrote in public forums about environmental problems (e.g., newspapers, blogs, Facebook, etc.).	In der Öffentlichkeit über Umweltprobleme zu schreiben (z.B. Zeitungen, Blogs, Facebook, etc.).

A five-point Likert scale ranging from 1 (=strongly disagree) to 5 (=strongly agree) or alternatively from 1(=never) to 5 (=always) for PEB is used. Items are used in the German language. N = number of items. PEB = pro-environmental behavior. Introduction text for Social and Personal Identity: everyone has certain ideas about their life and behavior. How do you see yourself (=Jeder Mensch hat bestimmte Vorstellungen von seinem Leben und Verhalten. Wie schätzen Sie sich ein)? Introduction text for Willingness to Pay and Consumer Behavior: there is both support and opposition to the environmental movement. What is your opinion (=Es gibt sowohl Unterstützung als auch Widerstand gegen die Umweltbewegung. Was ist Ihre Meinung)? Introduction text for Activist Behavior: how often do you do the following (=Wie häufig machen Sie bei Folgendem mit)?

#### Social identity (to climate protectors)

We measured social identity (to climate protectors) with an adjusted instrument by Cameron (2004). Test statistcs are estimated (ω = 0.88; *r_it_* = 0.69–0.82; *p_i_* = 0.24–0.27; item sample: I have a lot in common with other climate protester). The instruemnt is seen as excellent.

#### Environmental self-identity (personal identity)

The construct of environmental self-identity (personal identity) was operationalized using an instrument developed by van der Werff et al. (2013). The test statistics were evaluated, with ω = 0.85, *r_it_* = 0.65–0.77, and *p_i_* = 0.56–0.59. The item sample was also considered: I consider myself as a person that acts environmentally-friendly. The employed measurement instrument is considered to be of an acceptable standard.

#### PEB variables of willingness to pay, consumer and activist behavior

In our research, PEB measurement is distinguished by its classification into three subscales: willingness to pay, consumer behavior and activist behavior. Firstly, willingness to pay is measured using an instrument developed by Stern et al. (1999), which has been demonstrated to have good reliability (ω = 0.86; *r_it_* = 0.71–0.76; *p_i_* = 0.37–0.55; item sample: I would be willing to pay much higher taxes in order to protect the environment.). Secondly, consumer behavior was also measured using an instrument by Stern et al. (1999), with items that were adjusted and demonstrated adequate reliability (ω = 0.78; *r_it_* = 0.60–0.63; *p_i_* = 0.46–0.59; item sample: how often do you make a special effort to buy household chemicals such as detergent and cleaning solutions that are environmentally friendly?). Finally, thirdly, the degree of activist behavior is gauged by an adjusted scale of Schmitt et al. (2019) and exhibits an acceptable level of reliability (ω = 0.75; *r_it_* = 0.47–0.65; *p_i_* = 0.11–0.21; item sample: got involved with a club or group whose main aim is to preserve and protect the environment). Although two item difficulties, specifically “Handed out fliers or put up posters in public locations to raise awareness about environmental issues” and “Wrote in public forums about environmental problems (e.g., newspapers, blogs, Facebook, etc.),” indicate problematic values for the scale of activist behavior, the remaining coefficients are acceptable.

### Data analyses and missing values

We commence our analysis with an exploratory view of the data in the preliminary analysis chapter, utilizing SPSS (Version 29). Cohen (1988) proposed that Pearson’s *r* correlation be interpreted as follows: *r* = 0.10–0.29 represents a small correlation, *r* = 0.30–0.49 indicates a medium correlation, and *r* ≥ 0.50 signifies a large correlation. The evaluation of the normal distribution revealed that the skewness values were problematic, falling outside the range of −1 to +1 (Hair et al., 2014). A *p*-value of less than 0.05 (two-tailed) is deemed statistically significant.

We employ the structural equation modeling technique (SEM; Ullman, 2014) with the estimator maximum likelihood estimation with robust standard errors (MLM) using the package “lavaan” from Rosseel (2012) in the software R for testing our hypothesis. The model fit is evaluated using the cut-off criteria proposed by Hu and Bentler (1999), namely *RMSEA* ≤ 0.06, *CFI* and *TLI* ≥ 0.95, as well as *SRMR* ≤ 0.08. The comparison of the different significant effect sizes of social and personal identity in the three distinct PEBs is conducted using *z*-statistics and constraints within the model. Furthermore, we are testing the different effect sizes through bootstrapping (using 5,000 replications). In this approach, the null hypothesis of a different effect is rejected when the confidence interval of 95% does not include the value zero.

A total of 568 participants were included in the data collection process; however, there are instances where the data is incomplete. The proportion of missing values for variables ranged from 0 to 3.50% (*M* = 0.77%; *SD* = 0.71). For 535 participants (94.19% of the sample) and 99.27% of the existing values, no missing values were identified. A further analysis of missing values indicates that Little’s (1988) test of Missing Completely at Random (MCAR) was not significant, with a value of χ^2^ = 228.99, *df* = 225, and a *p*-value of 0.41. The data were found to be consistent with the assumption of missing completely at random (MCAR) (Peugh and Enders, 2004). Consequently, the missing data were replaced via multiple imputation, conducted using the chained equations function of the mice package in R with 100 imputations (Van Buuren and Groothuis-Oudshoorn, 2011).

## Results

### Preliminary analysis

Table 2 presents the descriptive statistics and correlations (*r*) of the scales employed in this study. The distribution of activist behavior is problematic. The skewness of the data is 1.23, which is indicative of a non-normal distribution. Nevertheless, attempts to transform this variable through the application of logarithms yielded unsatisfactory results, and thus, no transformation was deemed necessary.

**Table 2:** Descriptive statistics and bivariate correlations (*r*).

		1.	2.	3.	4.	5.
1. Social identity (to climate protectors)		0.88	0.46	0.58	0.42	0.38
2. Environmental self-identity (personal identity)		0.43	0.85	0.52	0.55	0.23
3. Willingness to pay		0.52	0.47	0.86	0.56	0.24
4. Consumer behavior		0.36	0.46	0.46	0.78	0.33
5. Activist behavior		0.36	0.24	0.24	0.28	0.75
	*M*	2.03	3.30	2.77	3.17	1.59
	*SD*	0.86	0.82	0.98	0.93	0.69
	*Skewness*	0.70	−0.34	−0.01	−0.28	1.23
	*Kurtosis*	0.14	0.32	−0.70	−0.44	0.93

*N* = 568. Reliability ω in diagonal. Likert Scale range from 1 (=strongly disagree/never) to 5 (=strongly agree/always). Manifest correlation below diagonal and latent correlation above diagonal.

The largest latent effect size of the correlations is observed for the association between social identity and willingness to pay (*r* = 0.58). Furthermore, there are notable large effect sizes for latent variables, including those associated with environmental self-identity and willingness to pay (*r* = 0.52), environmental self-identity and consumer behavior (*r* = 0.55), and willingness to pay and consumer behavior (*r* = 0.56).

### Results on the hypotheses

Structural equation modeling is employed for the analysis of our hypotheses. The goodness of fit indices of the estimation of our model indicate a good model fit (χ^2^ = 171.527; *df* = 80; χ^2^/*df* = 2.144; *p* < 0.001; *CFI* = 0.974; *TLI* = 0.966; *RMSEA* = 0.049; *SRMR* = 0.055). This result indicates that the theoretical assumptions are reflected in the data.

**Figure 1:**
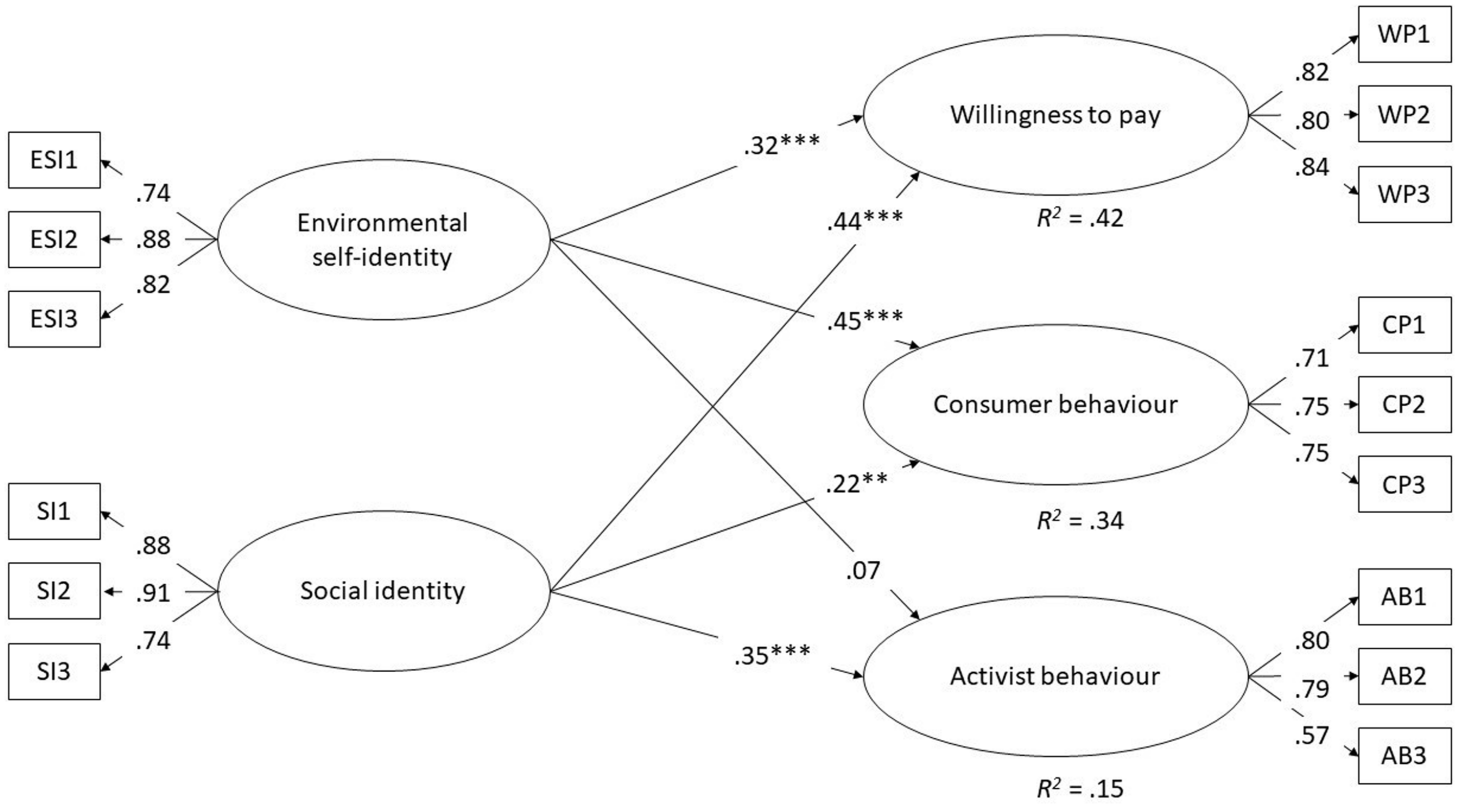
Results on analyzing two types of identity on the three components of pro-environmental behavior (*N* = 568). Figure shows standardized parameters using *p < 0.05, **p < 0.01, ***p < 0.001.

Figure 1 presents the results in graphical form. There is a significant association between environmental self-identity and both willingness to pay (β = 0.32; *p* < 0.001) and consumer behavior (β = 0.45; *p* < 0.001). Social identity is associated with willingness to pay (β = 0.44; *p* < 0.001), consumer behavior (β = 0.22; *p* = 0.001) and activist behavior (β = 0.35; *p* < 0.001). The proportion of the dependent variables that can be explained by the independent variables is greatest for willingness to pay (*R*^2^ = 0.42) and least for activist behavior (*R*^2^ = 0.15).

Table 3 presents a comparison of the effect size of personal and social identity on the three PEB components. The results indicate that there is no significant difference in the effect size between personal and social identity in the associations with willingness to pay (*b* = −0.067; 95% CI = [−0.285; 0.153]; *p* = 0.553). The effect size of personal identity is significantly higher than that of social identity (*b* = 0.287; 95% CI = [0.091; 0.490]; *p* = 0.005) in the context of consumer behavior. Conversely, for instances of activist behavior, there is a greater effect size for associations with social identity in comparison to personal identity (*b* = −0.189; 95% CI = [−0.358; −0.023]; *p* = 0.025).

**Table 3:** Tests of comparison from the different effect size between social identity and personal identity on three PEB components (*N* = 568).

	*b*	*SE*_Boot_	95% CI_Boot_	*p*
Δ effect sizes on willingness to pay	−0.067	0.113	−0.285, 0.153	0.553
Δ effect sizes on consumer behavior	0.287	0.102	0.091, 0.490	0.005
Δ effect sizes on activist behavior	−0.189	0.084	−0.358, −0.023	0.025

Δ = effect size comparison between social identity and environmental self-identity on the three PEB components. Differences of the effects are non-standardized parameters (b) using bootstrapping (5,000 replications). Positive values for non-standardized parameters (b) indicate a stronger effects size for personal values and negative values for non-standardized parameters (b) indicate a stronger effects size for social values on the three PEB components.

## Discussion

A substantial body of research indicates that identity plays a significant role in the decision-making process surrounding PEB. Nevertheless, the comparison of different identities in their effect size on PEB is still in its infancy, and further specific analyses, such as the cultural and educational background of the participants, are required. In order to address this research question, we conducted a study on the population of cooperative education in Germany.

Hypothesis 1, which posited a positive association between a personal identity with nature, as measured by environmental self-identity, and PEB, was partially supported. The analysis revealed significant positive associations between personal identity and two PEB variables: willingness to pay and consumer behavior. These findings are consistent with the theoretical framework of SCT and empirical research (Shadiqi et al., 2022; van der Werff et al., 2021). The study did not identify a significant relationship between personal identity and activist behavior. It would appear that general personal identity plays a relatively minor role in explaining activist behavior. As evidenced by studies such as Schmitt et al. (2019), politicized identity approaches have been found to influence activist behavior.

Hypothesis 2, which posits that a higher social identity with nature is positively associated with PEB, is confirmed. The results indicate that there are significant effects on all three dependent variables of PEB. These results corroborate the theoretical assumptions of the Social Cognitive Theory (SCT) and previous empirical findings (Chumg et al., 2019; Dono et al., 2010; Hehir et al., 2021; Schulte et al., 2021).

The empirical results offer partial confirmation of Hypothesis 3 and challenge the sweeping theoretical assumption of SCT that personal and social identity are of equal importance in the expression of the psychological process of self (Turner et al., 1994). Moreover, the general empirical findings that personal identity is more strongly associated with PEB than social identity (Udall et al., 2021; Vesely et al., 2021; Wang et al., 2021) cannot be supported. The empirical findings support the view that the effects on the detailed components of PEB are highly specific. It is not possible to make a general statement, as the analysis revealed the following specific findings: (1) there is no significant difference in the effect size on the PEB component of willingness to pay; (2) there is a stronger effect size for social values compared to personal values for activist behavior; and (3) there is a stronger effect size for personal values compared to social values for consumer behavior.

It is important to note that the degree of explained variance differs between the three dependent variables: willingness to pay (*R*^2^ = 0.42), consumer behavior (*R*^2^ = 0.34) and activist behavior (*R*^2^ = 0.15). This lack of explained variance indicates that important variables are missing from the model. Once more, the dependent variable of politicized identity (Schmitt et al., 2019) may be cited as an example of the activist behavior dependent variable. Haslam et al. (2022) offer an alternative explanation for the higher explained variance of the dependent variables and conclude, in line with the ‘loneliness epidemic’ and neoliberal ideology, that personal identity (in the form of individualism) is privileged over social identity.

The present work offers insights into the decision-making process of PEB. This allows us to elucidate the significance of diverse identities and their ramifications. The results presented here pertain to a postulated sophisticated population with a particular cultural and educational background (Kirbiš, 2023; Mónus, 2022). Research on this special population of cooperative students in Germany is important for several reasons. Firstly, the number of participants is increasing (Hofmann, 2023). Secondly, they have a lower social background compared to regular university students (Kramer et al., 2011). Thirdly, they have work experience and a contract with a company (Wild and Neef, 2019; Wild and Neef, 2023). Fourthly, they enroll in important academic disciplines of economics and engineering (Federal Statistical Office of Germany, 2022). Moreover, the situation in Germany is of significant importance due to its considerable economic influence, the decommissioning of nuclear power plants, and the fact that the Alliance 90/The Greens party is currently in government. Another noteworthy aspect of our research is the precise reliability (ω = 0.75–0.88) and satisfactory model fit of the estimated structural equation model (*CFI* = 0.974; *TLI* = 0.966; *RMSEA* = 0.049; *SRMR* = 0.055) in testing the hypotheses.

It should be noted that the research presented here is limited by a number of factors. The study employs a cross-sectional design with convenience sampling, which precludes the possibility of conducting causal analyses. However, research indicate a high degree of consistency in sustainable behavior even years apart (Puntiroli et al., 2022). Furthermore, the participants in the sample are drawn from a single higher education institution located in a single federal state in Germany. The data analyzed in this study were collected using self-report measures, which are considered problematic in the field of PEB due to the potential for measurement error or social desirability bias (Demetriou et al., 2015; Lange, 2023; Wild et al., 2023). In this research on PEB, we adopt a single theoretical perspective. Nevertheless, further theoretical frameworks from sociological theories, such as social interaction theory, or economic frameworks, such as extended rational choice assumptions including different intrinsic motives, should also be considered, as PEB is a multidisciplinary issue (Tian and Liu, 2022).

A generalization of the results of our study needs to be made carefully and depends on the specific cultural and educational context, which shows similarities to cooperative education in Germany. According to Wild and Neef (2019, 2023), a high workload for workplace training in a company should be integrated into the curriculum, which could also be the case for non-university education. Another important point is small learning groups, with a maximum of 30 students for the learning environment. We specify our sample to be students of economics and engineering, so this is another specific point for generalization. Another characteristic is that our participants have a contract with a company, which is another point of realization. One suggestion for further research could be a comparative study between co-operative higher education and vocational education and training. For the robustness of our results, international comparative studies should be carried out. This is the case for comparing non-WEIRD (western, educated, industrialized, rich and democratic) and WEIRD regions of the world.

Researchers discuss the association of socio-economic status on PEB (Eom et al., 2018). As Kramer et al. (2011) found that cooperative education students have lower social background compared to regular university students, this could be an explanation for the findings of this study. For example, people with low social background have different views and attitudes compared to people with high social background (Bourdieu, 1984, 1979; Coleman, 1988). This could be a starting point for analyzing the specific views of first generation students compared to other students. It would also be interesting to analyze the differences between traditional students and other students. Another way could be to control for social background variables in further research using statistical analyses, to develop a social background index for this area of research, or to use a more diverse sample.

The challenge for researchers is to reach a high level of knowledge through elaborated mediator and moderator effects between personal or social identity and PEB. Possible variables for mediator and moderator with initial empirical evidence associated with PEB could be moral (Schmitt et al., 2019) in terms of moral foundations theory (Graham et al., 2018), measured by Moral Foundations Questionnaire-2 (Atari et al., 2023), guilt or threat (Schmitt et al., 2019), which could influence human behavior. However, it must be noted that such interactions are complex and scientists need to consider the dynamics that drive PEB.

A number of suggestions have been put forth regarding the practical implications of strengthening PEB. In line with Vesely et al. (2021), it is emphasized that education, as well as virtual and real contact with nature, is essential for the development of political potential and identity, and ultimately motivation for PEB. This is evidenced by the fact that individuals are more likely to engage in PEB when they have a positive perception of their own group and a negative perception of other groups. Kirbiš (2023) proposes that the intervention should be implemented in vocational schools, as these individuals tend to have a lower educational background than their parents, which is associated with less pro-environmental attitudes. It is recommended that complex content on PEB be integrated into curricula at higher educational levels. One potential avenue for achieving this is through the use of mobile applications. Further ideas for practical implications on PEB are presented by Acquadro Maran et al. (2023) and Steg (2023) in their literature review. These include the use of media to inform people about the impacts of climate change and the reduction of energy consumption as a possible way forward It is recommended that further research be conducted into the aforementioned suggestions. Other potential strategies include the provision of financial and cash incentives, the implementation of negative reinforcements such as criticism and warnings, and the implementation of green training and development programs (e.g., workshops and seminars) for the management of environmental activities. Another aspect of factors influencing PEB is well-being and its relationship to the motivational theory of self-determination theory (Ryan and Deci, 2017). Kirbiš (2023) and Mónus (2022) show the influence of education on different aspects of PEB and suggest further research in this area. Another important aspect of this academic field is intersectionality (Singleton et al., 2022). We now know a little more about the factors associated with enhancing environmental behavior, which could be used by training companies, schools and universities for PEB. This knowledge could be incorporated into practical guidelines for training occupations, thus stimulating competency-based, sustainability-related teaching/learning arrangements.

## Conclusion

The objective of this study was to examine the extent to which personal or social identity affects PEB and to ascertain whether one of these two factors, the individual or the social factor, is more influential than the other. The study group comprised cooperative students in Germany. It is not possible to make a general statement, as the effect size of the associated factors on the three components of PEB under analysis shows considerable variation. The research question should be further developed with reference to other cultural and educational contexts, and the practical implications of the findings should be evaluated.

## Data Availability

The raw data supporting the conclusions of this article will be made available by the authors, without undue reservation.
